# Parental and Child Sleep: Children with Vision Impairment, Autistic Children, and Children with Comorbid Vision Impairment and Autism

**DOI:** 10.3390/brainsci14050485

**Published:** 2024-05-10

**Authors:** Nesli Guner, Jessica Antonia Hayton

**Affiliations:** Department of Psychology and Human Development, IOE, UCL’s Faculty of Education and Society, University College London, London WC1H 0AL, UK; n.guner.16@alumni.ucl.ac.uk

**Keywords:** sleep, parents, caregivers, children, autism, vision impairment, comorbid

## Abstract

Background: Parents report associations between children’s sleep disturbances and behaviors. Children with neurodevelopmental conditions (e.g., Williams Syndrome and autism) are consistently reported to experience increased sleeping problems. Sleep in children with vision impairment and children with a dual diagnosis of vision impairment and autism remains understudied. Methods: Our exploratory study compared sleep profiles in 52 children (aged 4–12 years) and their parents (*n* = 37), across four groups: children with vision impairment (VI; *n* = 9), autism (*n* = 10), comorbid vision impairment + autism (*n* = 6), and typically developing children (*n* = 27). Childhood sleep was measured using the parental report Childhood Sleep Habits Questionnaire and sleep diaries. Children’s cognitive functioning was measured using digit span, semantic, and phonemic verbal fluency measures. Parental sleep was measured via the Pittsburgh Sleep Quality Index and Epworth Sleepiness Scale. Results: Clinically disordered sleep was reported in all child groups (*p* ≤ 0.001), particularly children with VI + autism. Age, not sleep quality/quantity, predicted cognitive task performance in TD and autistic groups, but not in VI and VI + autism groups. The child’s diagnosis affected parental sleep, particularly in children with a dual diagnosis of VI + autism. Conclusions: All participants experienced problematic sleep to varying degrees. Those most affected were children and parents in the VI + autism group, suggesting that autism may be the main driver of sleep problems in our sample.

## 1. Introduction

Parental concern regarding their children’s sleep is well documented [1,2]. Problematic childhood sleep affects between 25 and 40% of children aged 1–5 years [3]. Although this is likely a conservative estimate of sleeping problems [4] it is consistently corroborated in the published literature. Childhood sleep problems are reported to be detrimental to cognition [5,6,7,8,9], behavior [10], social skills [11,12], and educational attainment outcomes [13,14]. In turn, this links to emotional and mood disorders, e.g., depression [15], hyperactivity and poor attention span [16,17,18], and poor relationships with peers and teachers [15,19]. Poor childhood sleep quantity (i.e., duration less than 10 h per night) in ages up to age 6 years old has been reported to detrimentally affect vocabulary development and is associated with inattentiveness, externalized behaviors, and hyperactivity [17,20]. Poor childhood sleep quality relates to anxiety and school phobia [21,22]. The evidence has subsequently suggested that the interplay between childhood sleep quality and quantity affects daytime functioning across social and cognitive domains.

Adults with vision impairment have predominantly been the focus of the literature to date [23,24,25,26]. Hayton et al. [27] reported that both typically developing children and children with vision impairment (VI; ranging from blindness to partial sight) had poor sleep quality and suboptimal sleep quantity on specific measures of sleep, indicating that sleeping problems were characteristic of the pediatric population, and not specific to diagnosis. However, the sample in [27]’s study did not extend to children with dual diagnoses of vision impairment and autism, nor any other comorbid condition. In children with vision impairment, comorbid conditions such as autism are reported in almost 50% of the population [28]. Evidence has suggested a prevalence of autism diagnosis ranging between 2 and 50% in the vision-impaired population [29,30,31,32]. However, little is understood regarding dual or separate diagnoses driving psychological and physiological effects on development [28]. To date, there are no reports of how the presence of VI + autism affects sleep, despite anecdotal interest from clinicians, parents, and educationalists.

Research concerning sleep in autism is more established. For example, [33] reported that autistic children who experience sleep problems are likely to demonstrate poorer daytime functioning, including academic attainment and social skill functioning. It is further argued that poor sleep can exacerbate the behavioral characteristics of autism, i.e., repetitive behaviors, and communication difficulties, ultimately worsening characteristics of the condition [34,35,36,37,38]. Disturbance to circadian rhythm (of which light is a key zeitgeber) has been reported in autistic children [39]. This is characterized by a delayed sleep phase (whereby the individual sleep pattern is delayed by 2 h or more from a typical sleep pattern), and an irregular sleep–wake pattern [39]. As the sleep–wake circadian rhythm is governed by light and dark, the severity of vision impairment (e.g., severe sight impairment and blindness) may affect the sleep–wake regulation [40]. For children with dual diagnoses of vision impairment and autism, circadian rhythm dysfunction and associated behavioral problems may be elevated.

Credible reports outline the similarities in behavioral characteristics in vision impairment and in autism [28,29,41,42,43,44]. Considerable behavioral overlaps have been reported in children with vision impairment and autistic children, relative to communication skills, social interaction, restricted symbolic play, and imitation (i.e., echolalic speech) [42,43,45,46,47]. These are understood as “stereotypic behaviors” in the field of autism and “Blindisms” in the vision impairment literature, which arguably may be resultant of a lack of “vision impairment friendly” autism assessment tools for children and young people with vision impairment. This is because the standardized autism assessment tools contain activities that rely on preserved/available vision to complete.

Despite the limited available literature regarding sleeping issues in children with VI and VI + autism, there is a body of work exploring the impact of poor sleep on cognitive functioning in developmental disorders and typically developing populations. Sleep is considered a critical process for healthy brain development and function [48,49,50]. Sleep quality rather than quantity is considered a predictor of cognitive functioning, whereby good quality yields positive effects [51]. The optimal quality and quantity of childhood sleep are associated with good performance in memory, attention, reasoning, and other cognitive processes [52]. Suboptimal sleep quality and quantity are associated with lower educational attainment, poor memory, reduced executive functioning, and behavioral and emotional maladjustment (e.g., [53,54,55]. However, much of the neurocognitive implications of sleep disturbance/sleep issues is resultant of correlational data, meaning that causation should not be assumed. Nevertheless, resounding evidence supports the notion that sleep disturbance affects cognitive functioning.

Sleeping problems in children with developmental disabilities may have cascading effects on the quality and quantity of parental sleep. Evidence shows that childhood sleep problems can extend to parental sleep disruption and have negative effects on family functioning [56,57]. The effects of childhood sleep problems on parental sleep are correlated with insufficient sleep quality, quantity, parenting stress, and parenting fatigue [58,59,60,61,62,63,64]. This can have adverse effects on parental physical health [65,66]. Maternal reports demonstrate how their child/ren’s sleep pattern directly impacts their own sleep and daytime functioning, leading to a lack of confidence in their ability to manage their child/ren’s sleeping patterns [64,67]. The negative effect on parental sleep is more pronounced in parents of children with developmental conditions, e.g., autism. The parents of autistic children are reported to have poorer sleep quality in comparison to the parents of typically developing children [68,69]. Correlations between the mothers’ descriptions of child/ren’s sleep issues and increased maternal physical and mental health issues have been reported; however, it is argued that these adverse effects can be mitigated by regulating mothers’ sleep quality [70].

Given that problematic sleep is evidenced in typically developing children, children with vision impairment, and autistic children, we hypothesize that children with comorbid vision impairment + autism (VI + autism) will also experience sleeping problems. Further, as parental sleep is credibly reported to be affected by their child/ren’s sleep, we expect similar patterns will occur. These overarching research questions, for child and parent sleep, guide our current study, which, to the best of our knowledge, is the first to explore the sleep profiles of children with VI + autism and their parents.

Specifically, we compared the sleep profiles and cognitive functioning of four groups of children: typically developing, vision impaired, autistic, and VI + autism, aged 4–12 years. We also compared parent sleep profiles, and if the diagnosis of the child affects parental sleep. We investigated the extent to which diagnosis may drive children’s sleep problems, cognitive functioning, and parental sleep.

## 2. Materials and Methods

### 2.1. Ethics

Ethics were subject to the BPS guidelines, reviewed and fully approved by UCL Institute of Education Student Research Ethics Committee. Fully informed parent and child consent/assent were gathered before participation in any aspect of the study. The parents and children were informed of their right to confidentiality, anonymity, and to withdraw. None of our samples withdrew.

### 2.2. Recruitment

The recruitment was primarily carried out via email and social media platforms throughout the UK. Autistic children were recruited from special educational needs schools and autism day-care centers. Children with VI or VI + autism were mainly recruited via Habilitation VIUK, forums, and correspondence with habilitation specialists. Potential participants’ legal guardians approached the researchers for in-depth study information (for both the parent and child participants). All participants (52 children and 52 primary caregivers/parents, one per child) were informed of their rights as participants and were invited to ask questions before giving consent. All study procedures were explained to the parents and their children either verbally and/or in writing. Data were gathered during the UK school term and before the COVID-19 pandemic. Community members were not involved in the conceptualization, design, or dissemination of findings.

The study inclusion criteria were that all child participants had a formal diagnosis of autism, VI, or comorbidity of VI + autism. The inclusion of typically developing (TD) children required that they were not under investigation for any suspected special educational needs and/or disabilities. Parents stated that all child participants had normal hearing (a fundamental element of engaging with the measures of cognitive functioning and following task instructions). Exclusion criteria for all child groups were that child participants were neither involved in a sleep intervention nor taking sleep medication, e.g., melatonin.

### 2.3. Measures

Data were collected from parents and children relative to the objectives of the study, detailed below.

### 2.4. Medical History Questionnaire (MHQ) (Child)

The parental report MHQ, developed at UCL Institute of Education, aided the contextualization of participant profiles, and understanding of pre-existing conditions. The MHQ measured individual characteristics and the current health, diet, and lifestyle habits of each child participant. The MHQ did not record ethnicity/race nor socioeconomic status/educational attainment levels.

### 2.5. Measures of Cognitive Functioning (Child)

Digit span test: a standardized subscale from WISC-IV [71] measures declarative/working memory in response to an auditory (spoken) stimulus, so it does not rely on vision. Each child listens to a line of digits increasing from 2 to 10 and repeats each line either in the order presented or in reverse order. Task instructions were followed. Each item contained two lines. To score, each line had a “trial score” of 0 or 1. The item score (i.e., the addition of two trial line values) was either 0, 1, or 2. The test was discontinued after scores of 0 on both trials of the item. The maximum raw scores were 16 for both digit span forwards and backwards, with a maximum total raw score of 32 (combining forward and backward scores). Raw scores are presented in Section 3.

Semantic and Phonemic Verbal Fluency [72]: Standardized measures that rely on auditory stimulus and do not involve vision. Each participant named as many animals as they could in one minute after an initial trial round (things you find in the kitchen). The phonemic verbal fluency test required each participant to list as many words starting with a certain letter in one minute (e.g., /f/, /a/ and /s/), without violating parameters such as names/repeated words. Scoring for both semantic and phonemic fluency was based on valid responses, i.e., within semantic category (animals) or within phonemic test parameters. Out-of-category semantic responses or repetitions were omitted from the score, as were phonemic responses that violated task parameters. Only raw scores are presented in the results.

### 2.6. Measures of Sleep (Child)

The Childhood Sleep Habits Questionnaire (CSHQ): The CSHQ screening test (45 items; [73]), is a standardized and reliable parental report measure of children’s sleep habits and sleep issues. The CSHQ yields acceptable internal consistency, test–retest reliability, and validity for community and clinical samples [73]. The questionnaire provides a total score along with eight subscales: bedtime resistance, sleep onset delay, sleep duration, sleep anxiety, night wakings, parasomnias, sleep-disordered breathing, and daytime sleepiness. The CSHQ requires parents to rate their child/ren’s sleeping habits against statements on a scale of 1–3 (1 = rarely, 2 = sometimes, and 3 = usually) based on a typical week for the child. The parents may also note whether each aspect addressed is causing issues for the child. Total score and subscale scores are calculated, as some items are reverse scored to highlight problem behaviors. Thus, a total score of 41 or higher indicates sleeping problems at the clinical level.

Sleep Diary: A five-night paper-based journal was kept by either the child or the parent to record bed- and wake-time and night wakings. An audio journal was offered to children, though none completed the diary in this format. The children were asked to keep their own diary where possible to maximize validity. The parents were asked to complete the sleep diary if their child was unable. Most parents kept the diary.

### 2.7. Measure of Sleep (Parent)

The Epworth Sleepiness Scale (ESS): a measure of parents’ sleepiness that comprises eight items/situations related to an individual’s propensity to become sleepy, ranging from 0, no probability of dozing, to 3, a high probability of dozing. The total score ranges from 0 to 24.

The Pittsburgh Sleep Quality Index (PSQI): a 19-item self-report questionnaire measuring different aspects of adult sleep, yielding a composite score in addition to seven component scores: subjective sleep quality, sleep latency, sleep duration, sleep efficiency, sleep disturbance, the use of sleep medication, and daytime dysfunction (with each scoring from 0 to 3). The total score ranges from 0 to 21, owing to the sum of the sub-scores in the standardized instructions.

Both the ESS and PSQI generally yield adequate/fair construct validity and internal consistency, though there is limited evidence regarding the test–retest reliability ([74,75], respectively). The standardized scores for both the ESS and PSQI are presented in Section 3.

### 2.8. Procedure

A complete study description and consent forms (for both parents and children) were emailed. The information and consent forms were prerequisites to participation and progression in the study. Testing was carried out during one season (Greenwich Mean Time, UK) to avoid potential sleep disturbance related to time difference. The parents and children first completed the online consent form. Next, a project pack with instructions was posted (pre-COVID-19) or e-mailed, based on the participants’ preference, containing the questionnaires and sleep diary. The questionnaires consisted of the MHQ, CSHQ, ESS, and PSQI. Upon completion, the families returned the forms via e-mail or pre-paid post. Last, contact was established (initiated by the parents or the research team) to conduct the digit span and verbal fluency tests with the children.

The verbal fluency and digit span measures were completed either in the participant’s home (pre-COVID-19) or via telephone. Face-to-face contact was not entirely necessary, thus facilitating a more open recruitment. Participating siblings in the same home were tested separately. The measures were conducted at a mutually agreed-upon time for the parents/children and the researchers. Accordingly, conducting the measures remotely or at home may have enhanced the ecological validity by providing a naturalistic research environment for the children and their parents. Additionally, the participants were notified of their rights regarding participation, including their right to withdraw, in accordance with ethical requirements.

### 2.9. Data Analysis

Data were analyzed using SPSS v.28. Participant demographics are presented first Next, descriptive statistics present the means and standard deviations for all the child and parent measures. Normality was tested using the Shapiro–Wilk test. A TD outlier was identified (on the PSQI measure) as were two TD outliers (on the bedtime measure). Once identified, these were removed from analysis to satisfy normality assumptions. The results are then organized according to child and parent groups.

Unless specified in the tables and analysis, child groups were aggregated into 2 groups: typically developing (TD) and non-typically developing (aggregating children with vision impairment (VI), autistic children (ASD), and children with VI + autism (VI + ASD). This was due to low sample sizes, and to broadly understand group differences prior to nuanced analysis.

Child data were analyzed using t-tests, correlations on age and outcomes (i.e., total scores for verbal fluency, digit span, CSHQ, and time in bed) followed by each cognitive measure, controlling for age, against time in bed, the number of sleep interruptions, and CSHQ subscale and total scores. *t*-tests were run on CSHQ subscales to explore differences between sleeping profiles of TD and non-TD groups. Extending from the *t*-test, a non-parametric subgroup analysis (Kruskal–Wallis) was run on VI, ASD, and VI + ASD total and subscale CSHQ scores to explore the differences between the groups. Correlations were then run on aggregated group data (TD and non-TD) regarding CSHQ subscales and sleep diary data, prior to the disaggregation of the non-TD group data for a nuanced interpretation.

Parent data were analyzed according to the same grouping as the child data, i.e., grouped into parents of TD children or parents of non-TD children (encompassing VI, ASD, and VI + ASD groups), owing to small sample sizes. A *t*-test was run to explore group differences in ESS and PSQI scores, followed by correlations between the parent (PSQI and ESS) and child (CSHQ) total and subscale scores.

## 3. Results

### 3.1. Participants

In total, 52 children and 52 primary caregivers/parents (one per child) gave informed consent to participate. The project had 100% participant retention; however, several participants failed to complete some measures. Of the recruited children, 19.23% (*n =* 10) did not participate in the measures of cognitive functioning. While all parents completed the Childhood Sleep Habits Questionnaire (CSHQ), 34.62% of the parents (*n* = 18) did not keep sleep diaries. Of the 52 parents who consented, 37 completed the Epworth Sleepiness Scale (ESS) and the Pittsburgh Sleep Quality Index (PSQI). The final sample for analyses was n = 52 children (n = 27 TD; n = 9 VI; n = 10 autistic (ASD); n = 6 VI + autism (VI + ASD); male n = 33; age range 4–12 years; M = 8.4 years) and n = 37 parents from across the UK. Table 1 shows the participant demographics of the final sample.

Table 2 shows that the autistic children had the poorest performance in the verbal fluency and digit span measures. Interestingly, on average, all children (TD, VI, ASD, and VI + ASD) were at or over the CSHQ threshold (>41) for clinical sleep problems. This is noteworthy regarding sleep quality considering that all children spent roughly the same time in bed. In relation to the parent questionnaires, it appears that the parents of children with VI + ASD have substantially more sleeping issues compared with parents of the remaining 3 groups (TD, VI, and ASD).

### 3.2. Results Part 1: Children’s Sleep

Statistically significant differences were reported on all CSHQ subscales between the TD and non-TD groups (Table 3).

Non-parametric subgroup analysis reported significant differences in the subscales of bedtime resistance *H*(2) = 7.149, *p* = 0.028 and sleep anxiety *H*(2) = 9.837, *p* = 0.007, indicating that the children with VI + ASD had more problematic sleep compared to the children with VI and children with ASD.

Significant correlations were reported in the TD children’s time in bed against CSHQ parasomnias r(17) = −0.601, *p* = 0.011 and CSHQ sleep-disordered breathing r(17) = −0.518, *p* = 0.033. Further, in the TD sample, sleep interruptions and CSHQ parasomnias were also positively correlated r(17) = 0.489, *p* = 0.047. In the non-TD group, sleep interruption and CSHQ sleep anxiety were positively correlated r(17) = 0.580, *p* = 0.015, as were sleep interruption and total CHSQ score r(17) = 0.508, *p* = 0.037.

In the VI group, significant associations were reported only in the relationship between sleep interruptions and CSHQ parasomnias r(4) = 0.962, *p* = 0.009. In the ASD group, two significant correlations were reported in the relationship between time in bed versus CSHQ parasomnias r(8) = −0.718, *p* = 0.045 and time in bed versus CHSQ total r(8) = −0.744, *p* = 0.34. In the VI + ASD group, two significant correlations were reported between time in bed versus daytime sleepiness r(4) = −0.986, *p* = 0.014, and time in bed versus the CSHQ total r(4) = −0.977, *p* = 0.023.

The TD children scored higher than the non-TD children on total verbal fluency scores t(40) = 2.19, *p* = 0.034, *d* = 0.68, and total digit span score t(40) = 2.95, *p* = 0.005, *d* = 0.92. Correlations (Table 4) demonstrated that age was positively correlated with verbal fluency and digit span performance in the TD children and autistic children, indicating that increased age leads to better task performance.

Although age appeared to predict verbal fluency and digit span performance in the TD and ASD groups, it did not predict sleep scores. In the TD group, correlations between time in bed and total digit span score were significant *r*(17) = −0.497, *p* = 0.042. In the VI group time in bed and semantic verbal fluency were correlated *r*(5) = −0.889, *p* = 0.044. In the ASD and the VI + ASD groups, time in bed and the CSHQ total were correlated *r*(8) = −0.744, *p* = 0.034 and *r*(4) = −0.977, *p* = 0.023, respectively. CSHQ subscales and cognitive performance were only significant in the VI + ASD group displayed in Table 5.

### 3.3. Results Part 2: Parental Sleep

The descriptive statistics for the parental ESS and PSQI scores (above, Table 2), showed that the parents of children with VI + ASD had considerably higher mean scores compared to the parents of TD, VI, and ASD children. A t-test exploring total PSQI scores reported that the parents of non-TD children had more problematic sleep (t(35) = −3.37, *p* = 0.002, *d* = 1.11). This was echoed in ESS scores (t(35) = −4.17, *p* ≤ 0.001, *d* = 1.35). The correlations between the CSHQ (child) total core and the total scores of the parent measures (ESS and PSQI) are presented in Table 6.

Table 7 and Table 8 show only those subscales that were statistically significantly related between the parent’s (PSQI) and children’s (CSHQ) responses.

Table 7 and Table 8 show that there was no overlap in related sleeping problems between the parents of TD children and parents of non-TD children.

## 4. Discussion

This exploratory study aimed to examine the differences in sleeping profiles and developmental conditions of four groups of children aged 4–12 years (children with VI, autistic children, children with VI + autism, and typically developing children), and the additional effects that childhood sleep could have on parental sleep.

Generally, our exploratory findings are consistent with the previous literature that reports greater sleep disturbance in children with a developmental disability (e.g., [7,8,76]), including autism (e.g., [77,78,79]) in relation to typically developing peers, and contradicted previous findings [27] where developmental condition (i.e., VI or typically developing) did not predict poor sleep outcomes. The CSHQ results further provide evidence that the comorbidity of VI + autism might be a stronger driving condition behind sleep problems and sleep disturbance in the pediatric population compared to a diagnosis of VI or autism alone.

Our findings aligned with our prediction that childhood/clinical diagnosis was significantly related to children’s sleeping problems recorded via the CSHQ and the sleep diary. Data showed that the children with VI, autistic children, and children with VI + autism demonstrated greater sleeping problems compared with the typically developing peers, though all the groups were at or above the CSHQ clinical threshold level for disordered sleep. The CSHQ scores indicated that the children with VI + autism had the greatest severity of sleeping problems among all four groups of children, while no group differences were found between the children with VI and autistic children. The TD children reported the fewest sleep interruptions compared with the other three groups. This finding may relate to the role of parental awareness in sleep hygiene. This means that parental awareness of the importance of sleep and the consequences of poor sleep may be unintentionally primed to overestimate sleeping problems in their child/ren [80].

Regarding sleeping profiles and cognitive functioning, the findings partially supported that of previous works [7,52,57,81,82,83]. Sleep interruptions were not correlated with cognitive task performance in subgroup analysis. Instead, chronological age appeared to be a stronger predictor of cognitive task performance. In partial support of previous work, the total CSHQ scores, time in bed, and cognitive task performance varied between all four groups. Inferring from time in bed, however, should be approached with caution. This is because some children may read, watch television, study, or engage in social media whilst in bed, which may disrupt circadian rhythm and sleep latency (time taken to fall asleep). As we could not use objective measures of sleep (e.g., actigraphy), we instead relied on sleep diaries, so we are unable to draw firm conclusions regarding sleep latency and “actual” sleep time (i.e., from falling asleep to waking up). Therefore, despite some statistical significance in our results, we are cautious with our claims regarding sleep quality, sleep quantity, and cognitive task performance. Age, as opposed to developmental conditions and sleep profiles, appears more convincing as driving task performance on the cognitive measures.

Our examination of the relationship between parent and child sleep showed that children’s sleeping problems did affect both parent groups on the ESS, and the non-TD group on the PSQI. Differences in the non-TD parents were more apparent, specifically in the parents of children with VI + autism. However, subscale analysis did show relationships between child and parent measures in both the TD and non-TD parent groups, namely on the PSQI. Our findings are consistent with previous research studies that demonstrated associations between children’s and their mothers’ sleep problems (e.g., [35,64,69]). Children’s average time in bed, however, was not related to the ESS and PSQI scores. This indicates that the CSHQ measure was a stronger predictor of parental sleep.

Owing to the small sample sizes in the non-TD parent group, only descriptive statistics could capture the subgroup differences. Of this, the ESS and PSQI scores were considerably elevated in the parents of children with VI + autism compared with the other three groups (VI, autism, and TD). This finding indicates that the dual diagnosis of the child may detriment parental sleep. However, as sibling data and parental lifestyle habits were not collected and as the sample was small in this group, we can only speculate that the dual diagnosis is responsible for poorer sleep in the parents of children with VI + autism. This means that more thorough parental measures would be needed to explore this phenomenon further.

The CSHQ results also showed that the mothers of autistic children had greater sleeping problems than the parents of children with VI and TD children. Contrary to expectations, group differences were not found between the mothers of children with VI and TD children and between the mothers of children with VI and autistic children. The lower parental scores for the TD, VI, and autistic groups could result from an increased parental/public awareness of sleep hygiene practices, as all three of these groups have previously been subject to research in this area.

Our findings supported our expectations that the mothers of children with comorbid VI + autism would experience a higher level of sleep problems than the mothers of the three other developmental groups. Our results also corroborated previous findings (e.g., [68,69]), where the mothers of autistic children experienced more sleeping problems compared with the mothers of TD children.

Despite our exploratory findings revealing sleep problems in all four groups of children and indicating that parental sleep may be affected by multiple diagnoses, our study is not without limitations. Small sample sizes in the parental groups meant we were unable to disaggregate the data to thoroughly explore the relationship between child and parental sleep according to child condition. Small sample sizes in the VI, autistic, and VI + autism groups also affected our ability to run a more detailed analysis of the sleeping profiles of children. Regarding our measures, we were limited to usable sleep diary data (specifically time in bed and sleep interruptions) and were unable to objectively measure sleep owing to limited resources. Thus, future work would benefit from a record of bed- and wake-time and using the objective measures of sleep (e.g., actigraphy). The latter is of importance and scientific interest, as discordance between the subjective (i.e., questionnaires) and objective (i.e., actigraphy and polysomnography) measures of sleep have been credibly reported [84,85,86]. Regarding children’s sleep, condition, and cognitive functioning, future work would also benefit from additional measures, e.g., the animal naming task (see [8]) as this would support links between sleep-dependent memory and/or memory consolidation, sleep quality, and sleep quantity.

Our exploratory work does, however, begin to address sleeping profiles in children with dual diagnoses of vision impairment and autism. Further, this project complements existing work regarding childhood sleep in children with vision impairment, autism, and typically developing children. As parental sleep was more affected in the VI + autism group, it is considered important to develop awareness of sleep hygiene practices to support the parents of, and children with, multiple diagnoses.

## 5. Conclusions

Our study supports the notion that sleeping problems are not subjective to children with developmental disabilities and are also apparent in typically developing children [87]. This is important for clinical practice. Given that optimal sleep quality and quantity are crucial for optimal physical, cognitive, and social–emotional development, the exploratory findings of our study should not be disregarded. Our findings support the case for an increased awareness of sleep hygiene in child and parent populations. The knowledge of good sleep practices including structured sleep routines can arguably benefit both parent(s) and child(ren) across multiple domains, including clinical practice. Timely detection and intervention addressing sleeping problems are required to impede the development of the potential escalating impacts of children’s sleep issues on parental sleep patterns. Therefore, the detailed subjective and objective assessment of parental sleep patterns, in future research studies and clinical practice, is also imperative, particularly within the pediatric setting. Finally, future studies regarding sleep and habilitation could be beneficial in developing interventions aimed at optimizing the sleeping routine for all children regardless of diagnosis (or a lack thereof), resulting in improved sleep and developmental outcomes throughout childhood, adolescence, and into adulthood. Suboptimal sleep quality and quantity remains a public health concern, affecting individuals worldwide, and so it is a priority to better understand sleeping profiles in targeted populations (including TD) which can then inform guidelines and interventions to promote and support sleep health strategies.

## Figures and Tables

**Table 1:** Participant demographics for child and parent sample.

	Total	TD	VI	ASD	VI + ASD
Number of child participants	52	27	9	10	6
Mean age in months (SD)	101.33 (26.58)	99.96 (25.71)	103.67 (30.15)	103.20 (27.78)	100.83 (29.93)
Age range in months	57–144	61–144	57–144	59–142	64–140
% males	63.5	63.0	66.7	60.0	66.7
Child Measures					
Verbal fluency; Valid *n*	42	20	6	10	6
Digit span; Valid *n*	42	20	6	10	6
CSHQ total; Valid *n*	52	27	9	10	6
S. Diary Bedtime; Valid *n*	34	17	5	8	4
S. Diary Sleep Interruption; Valid *n*	34	17	5	8	4
Parental Measures					
ESS; Valid *n*	37	20	2	10	5
PSQI; Valid *n*	37	20	2	10	5

**Table 2:** Descriptive statistics children’s scores for measures of cognitive functioning and sleep questionnaires and parental scores for measures of sleep quality and quantity.

	Mean Scores (Standard Deviation)			
	TD	VI	ASD	VI + ASD
Child Measures				
Verbal fluency total ^†^	36.65 (12.52)	33.83 (15.96)	24.90 (7.33)	28.00 (15.40)
Semantic total ^†^	15.85 (5.05)	15.00 (6.45)	11.50 (2.63)	12.33 (5.42)
Phonemic total ^†^	20.80 (7.90)	18.83 (10.47)	13.60 (4.57)	15.67 (10.19)
Digit span total ^†^	14.80 (2.38)	14.00 (3.23)	11.60 (2.22)	11.67 (3.98)
Forward ^†^	8.40 (1.63)	7.67 (2.06)	6.60 (1.50)	7.00 (2.60)
Backward ^†^	6.40 (1.23)	6.33 (1.86)	5.00 (0.94)	4.67 (1.50)
CSHQ total ^∫^	42.81 (5.38)	51.89 (9.47)	53.00 (9.26)	61.00 (9.51)
Bedtime resistance ^∫^	7.63 (1.82)	8.44 (3.20)	8.30 (0.94)	11.17 (1.32)
Sleep onset delay ^∫^	1.07 (0.26)	2.11 (0.92)	2.20 (0.78)	2.33 (0.51)
Sleep duration ^∫^	4.00 (1.33)	6.11 (2.52)	6.40 (2.17)	6.33 (1.86)
Sleep anxiety ^∫^	6.11 (2.19)	7.22 (2.63)	7.30 (0.82)	10.17 (1.16)
Night wakings ^∫^	3.67 (1.03)	4.89 (1.76)	4.90 (1.44)	6.33 (1.21)
Parasomnias ^∫^	8.78 (1.28)	9.22 (3.30)	10.50 (3.06)	10.50 (2.16)
Sleep-disordered breathing ^∫^	3.33 (0.62)	3.88 (1.35)	3.80 (0.63)	4.00 (0.63)
Daytime sleepiness ^∫^	11.59 (2.85)	14.22 (4.17)	14.10 (3.98)	14.83 (4.26)
Time in bed * (hours)	10.01 (0.70)	9.99 (0.95)	9.97 (0.07)	9.94 (0.18)
Sleep interruption *	0.2 (0.13)	0.64 (0.26)	0.96 (0.13)	1.30 (0.22)
Parent Measures				
ESS ^∫^	3.95 (2.76)	4.00 (2.83)	7.40 (2.76)	12.60 (2.70)
PSQI ^∫^	6.35 (3.62)	7.00 (1.41)	9.30 (2.87)	13.60 (2.70)

Note: * time in bed and sleep interruption scores are drawn from the sleep diary data, kept by the parents of the recruited children. ^†^ refers to raw scores on verbal fluency and digit span measures. ^∫^ refers to processed scores based on standardized scoring/task instructions.

**Table 3:** Mean scores (standard deviations), *t*-tests, and effect sizes for typically developing and non-typically developing group scores on the Childhood Sleep Habits Questionnaire.

Subscales					
	Non-TD (*n* = 25)	TD (*n =* 27)	t	*p*	*d*
Bedtime Resistance	9.14 (2.37)	7.63 (1.82)	−2.416	0.019	0.67
Sleep Onset Delay	2.20 (0.76)	1.07 (0.26)	−6.987	<0.001	2.00
Sleep Duration	6.28 (2.15)	4.00 (1.33)	−4.555	<0.001	1.28
Sleep Anxiety	7.96 (2.11)	6.11 (2.19)	−3.095	0.003	0.85
Night Wakings	5.24 (1.58)	3.67 (1.03)	−4.193	<0.001	1.18
Parasomnias	10.04 (2.92)	8.78 (1.28)	−2.043	0.046	0.56
Sleep-Disordered Breathing	3.88 (0.90)	3.33 (0.62)	−2.473	0.018	0.70
Daytime Sleepiness	14.32 (3.95)	11.59 (2.85)	−2.831	0.007	0.79
Total Score	54.52 (9.74)	42.81 (5.37)	−5.305	<0.001	1.50

**Table 4:** Subgroup correlations for age and outcome on total scores for verbal fluency, digit span, CSHQ, and time in bed.

	TD			VI			ASD			VI + ASD		
	*n*	r	*p*	*n*	r	*p*	*n*	r	*p*	*n*	r	*p*
Verbal fluency total	20	0.895	<0.001	6	0.972	0.23	10	0.975	<0.001	6	0.361	0.483
Digit span total	20	0.704	<0.001	6	0.525	0.285	10	0.932	<0.001	6	0.674	0.142
CHSQ total	20	−0.141	0.483	6	−0.206	0.594	10	0.066	0.855	6	−0.156	0.768
Time in bed	17	−0.171	0.513	5	−0.507	0.384	8	−0.073	0.864	4	0.449	0.551

**Table 5:** Correlations between CSHQ subscale scores and cognitive task performance in children with dual diagnosis of vision impairment and autism.

	VI + ASD		
CSHQ Subscales and Cognitive Functioning Measures	*n*	r	*p*
CSHQ bedtime resistance vs. verbal fluency total (phonemic and semantic)	6	0.830	0.041
CSHQ bedtime resistance vs. semantic verbal fluency total	6	−0.869	0.025
CSHQ sleep duration vs. verbal fluency total (phonemic and semantic)	6	−0.935	0.006
CSHQ sleep duration vs. semantic verbal fluency total	6	−0.904	0.013
CSHQ sleep duration vs. digit span total	6	−0.845	0.034
CSHQ total vs. verbal fluency total (phonemic and semantic)	6	−0.862	0.027
CSHQ total vs. semantic verbal fluency total	6	−0.833	0.039

**Table 6:** Correlations between CSHQ (child) total score and PSQI and ESS (parent) total scores.

	Parents of TD Children			Parents of Non-TD Children		
	*n*	r	*p*	*n*	r	*p*
CSHQ Total vs. ESS Total	20	0.447	0.48	17	0.679	0.003
CSHQ Total vs. PSQI Total	20	0.159	0.503	17	0.610	0.009

**Table 7:** Significant subscale correlations between PSQI and CSHQ scores of parents of typically developing children.

	Parents of TD Children		
Subscale Items (Parent Measure vs. Child Measure)	*n*	r	*p*
PSQI Daytime dysfunction vs. CSHQ sleep onset delay	20	0.479	0.033
PSQI Sleep duration vs. CSHQ parasomnias	20	0.531	0.016
PSQI Total vs. CSHQ parasomnias	20	0.463	0.040

**Table 8:** Significant subscale correlations between PSQI, ESS, and CSHQ scores of parents of non-typically developing children (children with vision impairment, autistic children, and children with vision impairment and autism).

	Parents of Non-TD Children		
Subscale Items (Parent Measure vs. Child Measure)	*n*	r	*p*
PSQI total vs. CSHQ daytime sleepiness	17	0.653	0.004
PSQI daytime dysfunction vs. CSHQ night wakings	17	0.505	0.039
PSQI sleep medication vs. CSHQ daytime sleepiness	17	0.516	0.034
PSQI sleep disturbance vs. CSHQ daytime sleepiness	17	0.624	0.010
PSQI sleep efficiency vs. CSHQ total	17	0.500	0.041
PSQI sleep efficiency vs. CSHQ daytime sleepiness	17	0.597	0.011
PSQI sleep latency vs. CSHQ total	17	0.490	0.046
PSQI sleep latency vs. CSHQ night wakings	17	0.494	0.044
PSQI sleep latency vs. CSHQ daytime sleepiness	17	0.564	0.018
PSQI subjective sleep quality vs. CSHQ daytime sleepiness	17	0.558	0.020
ESS vs. CSHQ sleep anxiety	17	0.514	0.035
ESS vs. CSHQ night wakings	17	0.542	0.025
ESS vs. CSHQ parasomnias	17	0.601	0.011
ESS vs. CSHQ daytime sleepiness	17	0.501	0.040

## Data Availability

The data are not publicly available due to the ethical approval stating that only processed data will be made available to preserve the anonymity and confidentiality of the participants. Data from the parent project are subject to the same constraints and any data collected in the parent project are not presented in this manuscript.
